# ID 343 - Incorporação do Projeto Terapêutico Singular por Equipes de Consultório na Rua: um estudo descritivo sob uma perspectiva de 10 anos

**DOI:** 10.5327/2237-9622.2025.v34s1.343

**Published:** 2025-11-25

**Authors:** Rafaela de Paula Sales, Thaís Barbosa de Oliveira, Aline Gonçalves Pereira

**Keywords:** Atenção Primária à Saúde, integralidade em saúde, vulnerabilidade em saúde

## Abstract

**Introdução:**

O Projeto Terapêutico Singular (PTS) configura-se como uma das tecnologias estratégicas de cuidado integral para a população em situação de rua (PSR), sendo elaborado, principalmente, pelas equipes de Consultório na Rua (eCR) no nível da Atenção Primária à Saúde (APS) no Brasil. As eCR utilizam o PTS na promoção de intervenções interdisciplinares com foco na autonomia e na reinserção social, fortalecendo vínculos familiares, comunitários e assegurando o acesso a direitos civis. Para a elaboração de PTS, as eCR realizam reuniões contando com articulação intersetorial e multidisciplinar. No entanto, as eCR enfrentam desafios na oferta dessa tecnologia, diante de disparidades geográficas e de estrutura assistencial. Portanto, este estudo objetivou descrever a realização de reuniões de eCR para a elaboração de PTS no Brasil, entre 2014 e 2023, identificando padrões temporais e regionais.

**Método:**

Trata-se de um estudo quantitativo descritivo sobre a realização de reuniões para elaboração de PTS por eCR entre os anos de 2014 e 2023. Os dados são de fonte secundária, extraídos da plataforma pública de Atividades Coletivas do Sistema de Informação em Saúde para a Atenção Básica (Sisab).

**Resultados:**

No período de 2014 a 2023, foram realizadas 29.218 reuniões para a elaboração de PTS por eCR no Brasil. O ano de 2023 registrou o maior número de atividades (18,37%, n=5.368) em comparação aos anos anteriores. Ao estratificar essas atividades por Grande Região, observou-se uma predominância no Sudeste (52,81%, n=15.431), seguido pelo Nordeste (36,95%, n=10.797). Em contrapartida, a Grande Região Norte apresentou a menor quantidade de registros (2,95%, n=863). Sob uma perspectiva histórica, entre os anos de 2014 e 2023, todas as Grandes Regiões apresentaram aumento de reuniões para elaboração de PTS por eCR, com uma variação percentual (VP) superior a 100%. No entanto, houve redução em alguns estados de três Grandes Regiões: no Nordeste, no estado da Paraíba (VP = -42,85%); no Centro-Oeste, no estado do Mato Grosso do Sul (VP = -450%); e no Sul, nos estados do Paraná (VP = -50,00%) e de Santa Catarina (VP = -78,94%).

**Conclusão:**

Os resultados deste estudo apontam para um crescimento constante na realização de reuniões para a elaboração de PTS pelas eCR no Brasil, entre 2014 e 2023, com predominância na região Sudeste do País. Essa concentração pode ser explicada pela maior quantidade de recursos, pela qualificação profissional e pelo elevado número de PSR na região, além do aumento da adesão dos municípios ao cofinanciamento federal das eCR nos últimos anos. Por outro lado, a Região Norte apresentou o menor número de reuniões, possivelmente devido à menor presença de equipes e aos desafios de mobilidade geográfica. As reduções de reuniões em alguns estados das Grandes Regiões Nordeste, Centro-Oeste e Sul podem estar ligadas a fatores locais, como dificuldades de implementação de políticas voltadas ao PTS ou falta de recursos humanos e estruturais. O ano de 2023, após a pandemia de covid-19, registrou o maior número de reuniões para PTS, o que pode refletir tanto o aumento da PSR quanto a necessidade de respostas mais estruturadas na atenção à saúde dessa população. Apesar do avanço proporcionado pelo uso do PTS para ampliar o acesso da PSR à APS, a queda no uso da estratégia em algumas regiões deve ser monitorada, considerando que o número de pessoas em situação de rua continua a crescer no Brasil.

